# Lifestyle and self-rated health: a cross-sectional study of 3,601 citizens of Athens, Greece

**DOI:** 10.1186/1471-2458-11-619

**Published:** 2011-08-04

**Authors:** Christina Darviri, Artemios K Artemiadis, Xanthi Tigani, Evangelos C Alexopoulos

**Affiliations:** 1 Postgraduate Course Stress Management and Health Promotion, School of Medicine, University of Athens, Soranou Ephessiou Str., 4, Postal code: GR-115-27, Athens (Greece

**Keywords:** self-rated health, health promotion, sleep, health inequalities, lifestyle, public health

## Abstract

**Background:**

Self-rated health (SRH) is a popular health measure determined by multiple factors. International literature is increasingly focusing on health-related behaviors such as smoking, dietary habits, physical activity, even religiosity. However, population-based studies taking into account multiple putative determinants of SRH in Greece are scarce. The aim of this study was to clarify possible determinants of SRH with an emphasis on the relationship between SRH and lifestyle variables in a large sample of urban citizens.

**Methods:**

In this one-year cross-sectional study, a stratified random sample of 3,601 urban citizens was selected. Data were collected using an interview-based questionnaire about various demographic, socioeconomic, disease- and lifestyle related factors such as smoking, physical activity, dietary habits, sleep quality and religiosity. Multivariate logistic regression was used separately in three age groups [15-29 (N = 1,360), 30-49 (N = 1,122) and 50+ (N = 1,119) years old] in order to identify putative lifestyle and other determinants of SRH.

**Results:**

Reporting of good SRH decreased with age (97.1%, 91.4% and 74.8%, respectively). Overall, possible confounders of the lifestyle-SRH relationship among age groups were sex, education, hospitalization during the last year, daily physical symptoms and disease status. Poor SRH was associated with less physical activity in the 15-29 years old (OR 2.22, 95%CI 1.14-4.33), with past or heavy smoking, along with no sleep satisfaction in the 30-49 years old (OR 3.23, 95%CI 1.35-7.74, OR 2.56, 95%CI 1.29-5.05, OR 1.79, 95%CI 1.1-2.92, respectively) and with obesity and no sleep satisfaction in the 50+ years old individuals (OR 1.83, 95%CI 1.19-2.81, OR 2.54, 95%CI 1.83-3.54). Sleep dissatisfaction of the 50+ years old was the only variable associated with poor SRH at the 0.001 p level of significance (OR 2.45, 99%CI 1.59 to 3.76). Subgroup analyses of the 15-19 years old individuals also revealed sleep dissatisfaction as the only significant variable correlated with SRH.

**Conclusions:**

Slight differences in lifestyle determinants of SRH were identified among age groups. Sleep quality emerged as an important determinant of SRH in the majority of participants.

## Background

Self-rated health (SRH) is among the most widely used health measures in epidemiological and medical research that it is based on the individual's perception of his/her health status rated in a four or five-point scale [1]. Self-assessment of health is considered a complex cognitive process that integrates health conceptions, comparisons with health-related references (such as earlier experiences, expectations, age etc) and cultural conventions [1]. Although, a definite explanation of this self-evaluation remains elusive, SRH is undoubtedly a robust marker of health-related quality of life and well-being. Most importantly, SRH is a strong predictor of morbidity, mortality and health services utilization [2-7]. This is attributed to the fact that SRH is a more inclusive measure than other direct health indicators, while it is hypothesized that it represents a conscious representation of biological processes, called the "interoception" concept [for review see ref. [1]].

Several determinants of SRH have been recognized, such as demographic, socioeconomic, behavioral, psychosocial and disease-related factors [8-15]. Currently, lifestyle health-related habits such as smoking, diet, sleep and exercise are gaining a growing attention in the international literature [16-24]. In general, individuals who follow a proactive lifestyle behavior report higher perceived health than those who do not [16-24]. Lifestyle determinants seem to vary among age groups, reflecting the correspondent differences of social health perceptions and requirements across lifetime [17,18].

A recent review of 56 studies states that "most investigations do not cover concomitantly the various aspects...of this multifaceted health measure" [25]. In the case of Greece, there are limited studies on the determinants of SRH [24,26-30]. Moreover, their majority failed to control for many putative determinants of SRH, focusing mainly on socio-demographic, lifestyle (such as religiosity, dietary habits and physical activity), and disease-related factors. Therefore, in this cross-sectional study, we try to estimate the relation of multiple socio-demographic, socioeconomic, lifestyle and disease-related factors with SRH in a sample of 3,601 urban citizens. To our knowledge, this is the first study in the Greek population that examines a wide range of lifestyle factors in the basis of other putative determinants of SRH.

## Methods

### Design

This is a cross-sectional study carried out in the city of Athens during the period of January 2006 to December 2007. The study was ethically and methodologically approved by The Research Committee of the Technological and Educational Institute of Athens. Eligible participants were residents of Athens of both sexes and over 15 years old. Participants consisted of a stratified random sample, representative of the Athens population. In more detail, households were randomly selected by assigning random numbers at first into different municipalities of Athens and secondly at 30 of their streets by using geographical maps of the area. Subsequently, 10 streets where again randomly selected. An open letter was then posted, informing residents of the buildings of the selected streets with regards to the date of visit and the purposes of the study. Trained health professionals performed the visits at the specific dates. 47% responded negatively or were absent for two consecutive visits. When needed, a new (third) appointment was scheduled, in order to increase response rates. All participants had to give his/her informed consent for entering the study. Interviews and somatometric characteristic measurements were performed at home by trained health professional students. Written informed consent was obtained before any measurement. Data collection was based on a face to face interview questionnaire, filled only by the interviewers. Sample size was determined by the one-year period of recruitment.

### Measurements

#### Dependent variable

SRH was based on the following statement: "In general, you would describe your health as...". There were five possible answers: "excellent", "very good", "good", "moderate" and "poor". For the main analyses good SRH was defined by the first three positive answers and poor SRH by the last two. Secondary subgroup analyses for "good" vs. "very good"/"excellent" health also took place.

#### Independent variables

Socio-demographic variables included age, gender and marital status. Age was divided into 3 equal groups: 15-29, 30-49 and 50+. Marital status was classified as married (or living with others) and unmarried (or living alone).

Socioeconomic status included four variables, namely education years, occupational status, social security coverage and house surface measured in square meters/person. Education years were divided into 2 groups: below 12 and above 12 years representing higher education. Occupational status was classified as employed and unemployed. Students, household-keepers and pensioners were included into the employed category, due to our specific interest in the category of unemployed people. Concerning, social security we created two main categories one including the major social security organization i.e. IKA (Social Insurance Institute) and one with all the other smaller agencies. House surface size (range 40 to 280 m^2^) per person (range 1 to 9) was divided into two equal groups with a cutoff point of 25.5 square meters/person. To our knowledge there are no available meaningful cut-offs for this variable and since checking for different ones is beyond the scope of this study, we chose to take the median value of 25.5 sqm/person.

Disease-related variables included health care use or hospitalization during the last year, disease status and daily physical symptoms. Hospitalization or health care use was based on a single question: "During the last year, did you need any medical attendance in a hospital setting? or "Have you received a medical prescription (temporal treatment) during last year". Only two possible answers were given: "yes" or "no". Disease status was calculated with the following open question: "Do you currently suffer from a condition needing regular medical treatment?" Individuals were asked to list their health problems and two doctors independently classified their answers as valid or not. Classification was as following: healthy (or disease free), one disease needing regular medication and finally comorbidity was defined as the presence of two or more diseases needing regular medication. Daily physical symptoms were based on a similar open question: "Is there any physical symptom hindering your everyday functioning during last year?" The majority of the answers provided were about musculoskeletal, gastrointestinal, headache and fatigue symptoms. We decided to keep the analyses coding simple as "yes" or "no", because the question gave emphasis on everyday functional impairment rather than the type of the symptom.

Lifestyle variables included smoking, dietary habits, sleeping patterns and religiosity.

Smoking was classified as no smoker, past smoker, light smoker (≤ 20 cigarettes per day) and heavy smoker (> 20 cigarettes per day).

Exercise was based on the question: "Do you work out more or less than 1 hour per week?" Answers were "more" or "less".

Dietary habits were recorded by asking the respondents about the number of glasses of alcohol they drink per week, and the number of days per week that they consume meat, fruits, vegetables, legumes and fish. Cut-offs were derived by the weekly guidelines about the Mediterranean diet: 0-1 days meat, 7 days fruits and vegetables, at least 1 day legumes, at least 2 days fish and 1 glass of any alcoholic beverage per day [31].

Each individual had his/her height and weight measured at the site of first encounter. All weighing machines were of the same brand name and model. Body mass index (BMI) was calculated as (Kg/m^2^), according to World Health Organization recommendations, and comprised of three categories for both men and women: normal (< 25 kg/m^2^), overweight (25-29.9 kg/m^2^) and obese (≥ 30 kg/m^2^).

Sleep satisfaction was assessed by answering the question: "Are you satisfied with the quality of your sleep". Answers were "yes" or "no".

Midday sleep was classified as "always", occasionally" and "no sleep", and it derived from a simple question asking participants if they sleep during midday.

Religiosity was explored by two parallel yes or no questions: (1) "Do you pray at least once a week?" (2) "Do you attend church ceremonies at least once a week?" Crosstabs analyses came up with two main subcategories: (1) those who responded yes to both questions and (2) those who responded no to both questions.

### Statistical analyses

All analyses were conducted separately for the three age subgroups. Descriptive measures of the categorical variables are presented as percentage values. Missing values did not exceed 3% in any of the independent variables. In table 1 comparison between subcategories for good SRH were made with chi squares tests. F values, degrees of freedom and Kendall's tau b coefficients (for significant only ordinal by ordinal comparisons) were also calculated and presented. Main multivariate logistic regression models were constructed by the socio-demographic, socio-economic and disease-related variables. We followed a stepwise method entering manually each variable beginning with sex (if sex was significant). All adjusted for sex variables that showed significance (Wald test) were entered in the final main model and then we checked for all possible interaction terms. Main models were initially fitted and each lifestyle variable was checked separately with main model by age group. Only statistical significant lifestyle variables were entered in the final model. No interactions were identified. Models are presented as odds ratios (OR) with 95% confidence interval (CI). We used Hosmer and Lemeshow test for goodness of fit and Nagelkerke R square values for the degree of "explanation" of the dependent variable (poor SRH) variance. The same method of modeling was applied for the subgroup analyses "good" vs. "very good"/"excellent" SRH. Statistical significance level for all analyses was set at 0.05. We further checked significant (p < 0.05) in the final multivariate models variables, performing a second multivariate logistic regression analysis, using a forward stepwise method with a 0.001 p level of significance for variable entry. Data were analyzed using the Statistical Package for the Social Science version 17.0 (SPSS, Inc., Chicago, IL, USA).

**Table 1:** Descriptive measures of independents variables by age groups and comparisons (chi square) for good Self-Rated Health (SRH)

Age groups	15-29 (N = 1,360)			30-49 (N = 1,122)			50+ (N = 1,119)		
*Variables*	%N	Good SRH (%)	F value (df)	%N	Good SRH %	F value (df)	%N	Good SRH (%)	F value (df)
***Socio-demographic***									
**Sex**			7.03(1)			16.81 (1)			22.74(1)
Male	44.3	98.5**		42.2	95.6**		46.5	81.5**	
Female	55.7	95.9		57.8	88.4		53.5	68.9	
**Marital status**			0.31 (1)			0.11 (1)			5.03 (1)
Married	32.8	97.5		41.6	91		38.8	78.6*	
Unmarried	67.2	96.8		58.4	91.8		61.2	72.4	
***Socio-economic***									
**Education (years)**			0.98 (1)			13.49 (1)			8.08 (1)
Less than 12	43.6	96.5		66.7	89.2**		83	73.3**	
More than 12	56.4	97.5		33.3	96		17	83.6	
**Occupational status**			0.98 (1)			0.41 (1)			0.91 (1)
Employed	94.7	97.2		98.2	91.6		99.5	74.7	
Unemployed	5.3	94.4		1.8	85		0.5	100	
**Health insurance agency**			0.02 (1)			2.29 (1)			1.02 (1)
All others	45.2	96.9		46.4	92.9		47.5	76.3	
IKA	54.8	97.2		53.6	90.2		52.5	73.5	
**Home surface/person**			0.0 (1)			3.91 (1)			0.01 (1)
> 25.5	46.2	97.1		45.5	93.3*		58.4	74.6	
≤ 25.5	53.8	97		54.5	89.9		41.6	75.1	
***Disease-related***									
**Health care use (last year)**			6.35 (1)			25.83 (1)			43.33 (1)
No	60.4	98.1*		46.1	96.1**		30.3	87.9**	
Yes	39.6	95.5		53.9	87.4		69.7	69.1	
**Daily physical symptoms**			4.47 (1)			6.35 (1)			30.19 (1)
No	22.5	99*		14.3	96.9*		9.6	97.2**	
Yes	77.5	96.5		85.7	90.5		90.4	72.4	
**Disease status**			23.36(2)			109.74 (2)			168.6 (2)
Healthy	65.8	98.7**		55.6	97.6**		29.8	93.3**	
At least one chronic disease	26	94.7		24.3	91.5		27.8	84.4	
Comorbidity	8.2	92.5		20.1	74.2		42.4	55.3	
***Food consumption***									
**Meat (times/week)**			0.58 (1)			0.41 (1)			0.46 (1)
0-1	21.4	96.2		16.9	90		27	73.2	
2 or more	78.6	97.3		83.1	91.7		73	75.4	
**Vegetable (times/week)**			0.0 (1)			0.01 (1)			0.0 (1)
7	28.5	97.2		35.2	91.6		43.8	74.9	
6 or less	71.5	97		64.8	91.3		56.2	74.7	
**Fruit (times/week)**			1.39 (1)			0.0 (1)			0.79 (1)
7	35	97.9		46.9	91.4		59.5	75.8	
6 or less	65	96.6		53.1	91.4		40.5	73.3	
**Legume (times/week)**			0.01 (1)			8.9 (1)			7.14 (1)
At least 1	85.7	97		93.9	92.1**		94.5	75.7**	
No consumption	14.3	97.4		6.1	80.9		5.5	59.7	
**Fish (times/week)**			2.4 (1)			1.49 (1)			0.3 (1)
At least 2	18	98.8		21.7	89.3		34.1	75.9	
1 or no consumption	82	96.7		78.3	92		65.9	74.2	
***Lifestyle***									
**Smoking**			7.26 (3)			8.26 (3)			8.47 (3)
No	50.7	98.3		38.3	94*		54.3	73.5*	
Past	2.8	94.7		8.2	88		17.4	69.7	
Light (≤ 20 cigarettes)	37.4	95.9		34.3	91.4		17.8	80.9	
Heavy (> 20 cigarettes)	9.1	96		19.2	87.9		10.5	79.5	
**Exercise (> 1 hour/week)**			2.26 (1)			0.03 (1)			0.0 (1)
Yes	64.9	97.6		56	91.2		52.9	74.8	
No	35.1	96		44	91.7		47.1	74.8	
**Alcohol (glasses/week)**			0.02 (1)			0.65 (1)			8.85 (1)
7 or less	88.1	97		80.7	91.1		81.1	72.9**	
Above 7	11.9	97.5		19.3	93.1		18.9	83	
**BMI categories**			2.43 (2)			9.27 (2)			14.4 (2)
Normal	80	97.3		48.6	92.8*		31	79.4**	
Over weight	16.7	96.9		39	92		46.9	76.4	
Obese	3.3	93.3		12.4	84.8		22.1	66	
**Sleep satisfaction**			4.51 (1)			25.45(1)			66.43 (1)
Yes	69	97.8*		70	94.3**		73.6	81.2**	
No	31	95.5		30	84.9		26.4	56.9	
**Midday sleep**			4.72 (2)			5.01 (2)			2.82 (2)
No	34	96.1		32.3	89.5		22.9	76.6	
Always	11.8	95.6		19.3	89.9		33.5	71.7	
Occasionally	54.2	98		48.4	93.4		43.6	76.2	
**Religiosity**			0.0 (1)			3.24 (1)			1.78 (1)
Yes	4	96.4		5.8	84.6		23.5	71.5	
No	96	97.1		94.2	91.9		76.5	75.8	

**p < 0.01 *0.01 ≤ p < 0.05 (chi square)

## Results

### Sample description and univariate analyses for good (good/very good/excellent) SRH

The number of respondents in each age group was 1,360 for 15-29 years old, 1,122 for 30-49 years old and 1,112 for 50-96 years old. In the first age group there were 602 males and 758 females, in the second 473 males and 649 females and in the third 520 males and 599 females (Table 1). Good SRH was reported by 74.8% of elders compared to 97.1%, 91.4% of the first two age groups (p < 0.0001).

Table 1 shows descriptive measures of the independent variables' subcategories for each age group. For all respondents better SRH was reported by males, individuals with no hospitalization or health care use during the last year and the non diseased. Independently from age a higher prevalence of good SRH was also reported by people who reported satisfaction with their sleep quality. In the middle-aged group significant higher prevalence of good SRH was also reported by highly educated, resident with more than 25.5 m2/person, non smokers, legume consumers for at least 1 day/week and with normal BMI (Table 1). Higher education, smoking status, alcohol use, consumption of legumes and BMI exhibited significance in the oldest age-group.

Kendall's tau b for ordinal significant only variables (with ordinal SRH from excellent to poor) by age groups were the following: 1) 15-29 years old: disease status 0.13 (SE 0.03, p < 0.0005), 2) 30-49 years old: disease status 0.28 (SE 0.03, p < 0.0005), smoking 0.07 (SE 0.03, p 0.017), BMI 0.07 (SE 0.03, p 0.037) and 3) 50+ years old: smoking -0.05 (SE 0.03, p 0.071), BMI 0.1 (SE 0.03, p 0.001). The above values are in accordance with the chi-square results in table 2.

**Table 2:** Main and final multivariate logistic models for poor Self-Rated Health (good SRH = reference) with socio-demographic, socio-economic and disease- and lifestyle-related variables among age groups

15-29 years old^a^			30-49 years old^b^			50-96 years old^c^		
Variables in main model 1	OR (95%CI)	p	Variables in main model 2	OR (95%CI)	p	Variables in main model 3	OR (95%CI)	p
**Sex**			**Sex**			**Health care use (last year)**		
Male	1		Male	1		No	1	
Female	2.26 (1.04 to 4.92)	0.039	Female	2.18 (1.24 to 3.82)	0.007	Yes	2.1 (1.42 to 3.1)	< 0.001
**Health care use (last year)**			**Education (years)**			**Daily physical symptoms**		
No	1		Less than 12 years	1		No	1	
Yes	1.99 (1.01 to 3.92)	0.047	More than 12 years	0.42 (0.23 to 0.77)	0.005	Yes	6.72 (2.06 to 21.91)	0.002
**Disease status**			**Health care use (last year)**			**Disease status**		
Healthy	1		No	1		Healthy	1	
One chronic disease	3.76 (1.74 to 8.11)	0.001	Yes	2.21 (1.26 to 3.9)	0.006	One chronic disease	2.17 (1.26 to 3.72)	0.005
Comorbidity	4.85 (1.87 to 12.56)	0.001	**Disease status**			Comorbidity	8.09 (5.0 to 13.09)	< 0.001
			Healthy	1				
			One chronic disease	3.55 (1.74 to 7.25)	0.001			
			Comorbidity	10.07 (5.24 to 19.37)	< 0.001			
Model characteristics								
R square (Nagelkerke)	0.098			0.252			0.255	
Goodness of fit Test Sig.	0.767			0.311			0.887	
(Hosmer and Lemeshow)								
**Final model (variables adjusted for model 1)^d^**	**OR (95%CI)**	**p**	**Final model (variables adjusted for model 2)^e^**	**OR (95%CI)**	**p**	**Final model (variables adjusted for model 3)^f^**	**OR (95%CI)**	**p**
**Exercise (> 1 hour/week)**			**Smoking**			**BMI categories**		
Yes	1		No	1		Normal	1	
No	2.22 (1.14 to 4.33)	0.02	Past	3.23 (1.35 to 7.74)	0.009	Overweight	1.32 (0.9 to 1.93)	0.153
			Light (≤ 20 cig.)	1.45 (0.8 to 2.62)	0.222	Obese	1.83 (1.19 to 2.81)	0.006
			Heavy (> 20 cig.)	2.56 (1.29 to 5.05)	0.007	**Sleep satisfaction**		
			**Sleep satisfaction**			Yes	1	
			Yes	1		No	2.54 (1.83 to 3.54)	< 0.001
			No	1.79 (1.1 to 2.92)	0.019			
Model characteristics								
R square (Nagelkerke)	0.12			0.28			0.30	
Goodness of fit Test Sig.	0.757			0.709			0.116	
(Hosmer and Lemeshow)								

^a ^Eliminated variables: marital status, education years, occupational status, health insurance agency, home surface, daily physical symptoms

^b ^Eliminated variables: marital status, occupational status, health insurance agency, home surface, daily physical symptoms

^c ^Eliminated variables: marital status, occupational status, health insurance agency

^d ^Eliminated variables: smoking, alcohol, meat, vegetable, fruit, legume and fish consumption, BMI, sleep satisfaction, midday sleep, religiosity

^e ^Eliminated variables: exercise, alcohol, meat, vegetable, fruit, legume and fish consumption, BMI, midday sleep, religiosity

^f ^Eliminated variables: smoking, exercise, alcohol, meat, vegetable, fruit, legume and fish consumption, midday sleep, religiosity

1 = reference category

p < 0.05 for multivariate logistic regression analyses.

### Multivariate analyses for poor/moderate vs good/very good/excellent SRH among age groups

Table 2 shows the ORs and 95% confidence intervals for poor SRH in three multivariate models for each age group. Among young participants (< 30 years), females, sufferers from one or more chronic diseases, and individuals who recently used health care or were hospitalized reported poor health (Table 2). In the middle-age group educational level was additionally retained to the model (Table 2). In elders only disease or symptom related variables significantly affected poor SRH reporting (Table 2). Comorbidity in all age-groups yields higher ORs than one disease alone. In the final models, the significant lifestyle variables included the followings: In the age group 15-29, reporting exercise for less than 1 hour per week increased the possibility of reporting poor SRH (OR 2.22, 95%CI 1.14 to 4.33). In the middle-aged individuals, past or current heavy smoking, along with poor sleep satisfaction were associated with increased report of poor SRH (ORs 3.23, 2.56, and 1.79, respectively) (Table 2). Finally, in the older age group, both BMI indicating obesity and poor sleep satisfaction were associated with poor SRH (Table 2).

In the final multivariate models constructed by the forward stepwise method with 0.001 p level of significance, all adjustment variables remained significant for all age groups, while only sleep dissatisfaction was related with poor SRH in the 50+ years old individuals (OR 2.45, 99%CI 1.59 to 3.76).

### Multivariate analyses for good vs very good/excellent SRH among age groups

Additionally, we have compared the subgroups reporting "good" versus "very good or excellent SRH" (data not presented in Table). These analyses revealed that the same pattern was pertained across each age group, although findings exhibited slightly lower strength of associations. In the first group (15-29 years), females (OR 1.55, 95%CI 1.16 to 2.06), unmarried participants (OR 1.47, 95%CI 1.08 to 2.0), who have used health services in the past year (OR 1.45, 95%CI 1.1 to 1.92), who reported at least one chronic disease or comorbidity (OR 1.17, 95%CI 0.85 to 1.61, and 3.31; 95%CI 2.13 to 5.16) and those sufferers physical symptoms (OR 3.34; 95%CI 2.13 to 5.24)) reported worst (i.e. good in this case) SRH. Regarding lifestyle variables, individuals reporting no sleep satisfaction (OR 2.59, 95%CI 1.92 to 3.49) and who consumed vegetables less than 7 times a week (OR 2.05, 95%CI 1.43 to 2.93) also reported worst SRH.

In the middle age group (30-49 years old), females (OR 1.54, 95%CI 1.17 to 2.03), use of health care services in the past year (OR 1.63, 95%CI 1.24 to 2.16), physical symptoms (OR 2.4, 95%CI 1.56 to 3.7) and one chronic disease or comorbidity (OR 1.47, 95%CI 1.06 to 2.03 and OR 2.13, 95%CI 1.45 to 3.12 respectively) were associated with poorer SRH, while higher education was significantly related to better SRH (OR 0.67, 95%CI 0.5 to 0.9). Regarding lifestyle variables, lack of sleep satisfaction was the only variable associated with worse levels of SRH (OR 1.55, 95%CI 1.15 to 2.21).

In the oldest group, health care use in the past year (OR 1.56, 95%CI 1.14 to 2.12), IKA insurance (OR 2.19, 95%CI 1.38 to 3.49), one chronic disease (OR 2.15, 95%CI 1.31 to 3.51), comorbidity (OR 4.94; 95%CI 2.83 to 8.65), daily physical symptoms (OR 1.79, 95%CI 1.16 to 2.79), and from lifestyle factors only sleep dissatisfaction (OR 2.16, 95%CI 1.46 to 3.25) contributed in reporting lower levels of SRH.

## Discussion

The objective of this cross-sectional study was to clarify putative determinants of SRH with an emphasis on the relationship between SRH and lifestyle factors in a large sample of urban citizens. As expected, female gender and more consistently disease status and healthcare use were associated with poor SRH, while significant variability of SRH putative determinants across age groups was monitored.

In the younger group, individuals reporting exercise less than one hour per week have poorer SRH, implying a particular awareness of the role of exercise on health among young people.

Concerning the middle age group, people with lower education level, past or heavy smokers and individuals dissatisfied with their sleep quality reported poorer SRH. Education was significant only in this age group, probably due to its higher correlation with putative socio-economic (and employment related) inequalities. Past and current heavy smokers seem to consider smoking habit more relevant to their health, possibly representing past reasons for quitting and worries of excessive smoking, respectively.

In the 50+ years old group, daily physical symptoms, increased BMI score and low sleep satisfaction, were associated with poorer SRH. Interestingly, the role of gender was not significant in the elders while BMI emerged as a new SRH determinant. The most consistent correlation was that of sleep dissatisfaction and poor SRH, even in younger people when subgroup analyses (good vs very good/excellent SRH) was performed. Moreover, sleep dissatisfaction was the only lifestyle variable associated with poor SRH in elders, even when higher level of significance was applied (0.001).

Overall, the explained variances of SRH from the youngest to the oldest age group were 12%, 28% and 30% respectively. The lower explained variance in the youngest group implies that other than the measured factors may determine SRH in younger people. We hypothesize that the lack of measured psychosocial factors, related mainly to interpersonal relationships or academic performance could account for this low percentage of explained variance. To test if such an assumption could be true, we repeated multivariate analyses separately for 15-19 years old and 20-29 years old people, considering that the younger group refers mainly to students of secondary education, while the second one represents students at university or labor force. For the first group, only sleep dissatisfaction correlated with poor health (OR 3.68, 95%CI 1.09 to 12.41, R square 0.05) and for the second one, variables were the same with the initial (e.g. 15-29 years old) group with little R square increase (explained SRH variance equal to 17%).

In the literature, there are a vast number of similar studies that differ in respect of participants' characteristics and measurement issues, a fact that hinders a complete "face-to-face" comparison with our own. However, our main findings are quite similar with those of the most pertinent previous studies, albeit in different cross-cultural settings, that have shown, the significant relations of SRH with smoking [17,18,20,22], regular physical exercise [16,17,19,22,23,28,32,33] and obesity [17,34,35]. Finally, sleep quality, although differently assessed in various studies, seems to have an important relationship with SRH [16,17,21,33,36,37]. Comparing our work to these pertinent studies, major measurement discrepancies were detected for exercise and sleep quality, although this did not affect their role in determining SRH. Overall, for exercise most surveys used validated questionnaires, sets of various questions on everyday physical activity or impressions about the level of fitness, while our measurement was more simplistic (more or less than one hour per week) mainly based on the usual recommendations by Greek physicians. The same straight-forward pattern describes sleep satisfaction, while other studies used validated questionnaires or questions relevant to sleep quality (e.g. hours of sleep).

On the contrary, we did not found any relationship between dietary habits or alcohol intake and SRH supported by previous studies [17,18,22]. We hypothesized that cross-cultural differences might account for these differences [38]. We should note, however, that in other studies dietary habits were measured according to servings per day for each food category [18] or in the context of health behaviors (choices) [22], while we used a more crude measure of consumption (days per week), which it might has affected our results. We did not found significant relationship between religiosity and SRH as few other studies also did [27,39]. We used two arbitrary open questions to address both extrinsic (church attendance) and intrinsic (praying) religiosity, which is not common among other studies. Religiosity is a complex variable, which should incorporate not only praying, or attendance to ceremonies, but also psychosocial variables pertinent to social networks and social recourses or support [40]. As such, religiosity could be a marker of various social-related factors and should be regarded with caution. Perhaps spirituality may be taken into account in future studies [41].

It is acknowledged that this study has a number of limitations. Firstly, cross-sectional analyses cannot infer causality among measured factors. Secondly, measurements are mainly based on self-report increasing the likelihood of information and recall bias. Seasonal variation in responses during the one year period of the study may contribute to information bias. Moreover, inter-interviewer variability and issues with regard to social communication (e.g. social desirability) may more likely introduce measurement bias than simple questionnaire administration, but interviews have the advantage of minimizing missing values, which was the case in our study. Thirdly, low participation rate (53%) could introduce a selection bias and impair generalizability, although no cluster of low participation rate among regions was noted. Fourthly, dietary cut-offs were made according to the guidelines of Mediterranean diet without including information about the level of individuals' awareness to these directions. As written earlier in the introduction, SRH is subject to individual's perceptions and expectations, so a poor informative status about diet could deviate the association of this lifestyle matter with SRH. Finally, we did not include psychosocial variables which could be putative mediators or moderators of the lifestyle-SRH relationship.

## Conclusion

This is the first population-based survey in Greece include a plethora of lifestyle and other factors in respect to self rated health. Our study shows that - adjusted for other well known SRH related factors - lack of regular exercise, obesity, past or heavy smoking and mainly sleep dissatisfaction were related with poor SRH in a randomly selected urban population. Low explained variances of SRH, especially in the younger group, may underline the need for psychosocial measurements in future studies. The fact that lifestyle factors were associated with SRH, underlines the need to be incorporated in self-awareness and health-promotion context.

## List of abbreviations

SRH: self-rated health; OR; odds ratio; CI: confidence intervals.

## Competing interests

The authors declare that they have no competing interests.

## Authors' contributions

The conception and design of this study was made by CD. For data collection XT was responsible. Under the guidance of ECA, AKA performed the statistical analyses and wrote the first draft of the article. All authors contributed equally to the interpretation process. CD and ECA revised the manuscript. All authors read and approved the final manuscript

## Pre-publication history

The pre-publication history for this paper can be accessed here:

http://www.biomedcentral.com/1471-2458/11/619/prepub
